# Supplementary material for: High risk of patient self-inflicted lung injury in COVID-19 with frequently encountered spontaneous breathing patterns: a computational modelling study
Source: Ann Intensive Care. 2021 Jul 13;11:109. doi: 10.1186/s13613-021-00904-7 (PMC8276227; doi:10.1186/s13613-021-00904-7)
Supplement: Supplementary file 1 — Additional file 1. Full description of computational simulator, additional results and figures. [file 13613_2021_904_MOESM1_ESM.docx]

**High risk of patient self-inflicted lung injury in COVID-19 with frequently encountered spontaneous breathing patterns: a computational modelling study**

**Additional File**

The online data supplement for this paper contains additional material that could not be included in the main text due to space limitations. The following sections describe in detail the simulation model employed in the paper.

Table of Contents

[Section 1: ICSM Pulmonary Model 2](#_Toc75781199)

[Section 2: Data for the population of 10 patients at baseline 12](#_Toc75781200)

[Section 3: Modelling spontaneous ventilation 13](#_Toc75781201)

[Section 4: Modelling Covid-19 Acute Respiratory Failure 14](#_Toc75781202)

[Section 5: Effect of Respiratory effort on compartmental volumes 15](#_Toc75781203)

[References 18](#_Toc75781204)

##

| Section 1: ICSM Pulmonary Model **** |  |
| --- | --- |
| **Figure S1.1 Diagrammatic representation of the pulmonary model** |  |
|  | |

The model employed in this paper has been developed over the past several years and has been applied and validated on a number of different studies (1-8). The model is organised as a system of several components (see Fig S1.1), each component representing different sections of pulmonary dynamics and blood gas transport, e.g. the transport of air in the mouth, the tidal flow in the airways, the gas exchange in the alveolar compartments and their corresponding capillary compartment, the flow of blood in the arteries, the veins, the cardiovascular compartment, and the gas exchange process in the peripheral tissue compartments. Each component is described as several mass conserving functions and solved as algebraic equations, obtained or approximated from the published literature, experimental data and clinical observations. These equations are solved in series in an iterative manner, so that solving one equation at current time instant $(t_{k})$ determines the values of the independent variables in the next equation. At the end of the iteration, the results of the solution of the final equations determine the independent variables of the first equation for the next iteration.

The iterative process continues for a predetermined time, *T*, representing the total simulation time, with each iteration representing a ‘time slice’ *t* of real physiological time (set to 10 ms). At the first iteration$(t_{k}, k=0)$, an initial set of independent variables are chosen based on values selected by the user. The user can alter these initial variables to investigate the response of the model or to simulate different pathophysiological conditions. Subsequent iterations ($t_{k}= t_{k-1}+t$) update the model parameters based on the equations below.

The pulmonary model consists of the mechanical ventilation equipment, anatomical and alveolar dead space, anatomical and alveolar shunts, ventilated alveolar compartments and corresponding perfused capillary compartments. The pressure differential created by the mechanical ventilator or inspiratory muscles (i.e. when modelling spontaneous breathing) drives the flow of gas through the system. The series dead space (SD) is located between the mouth and the alveolar compartments and consists of the trachea, bronchi and the bronchioles where no gas exchange occurs. Inhaled gases pass through the SD during inspiration and alveolar gases pass through the SD during expiration. In the model, an SD of volume 60ml is split into 50 stacked layers of equal volumes ($N_{SD}$ = 50). No mixing between the compartments of the SD is assumed.

Any residual alveolar air in the SD at the end of expiration is re-inhaled as inspiration is initiated. This residual air is composed of gases exhaled from both perfused alveolar compartments (normal perfusion) and the parallel dead space (PD) (alveolar compartments with limited perfusion). Therefore, the size of dead space (SD and PD) can have a significant effect on the gas composition of the alveolar compartments.

The inhaled air is initially assumed to consist of five gases: oxygen (O_2_), nitrogen (N_2_), carbon dioxide (CO_2_), water vapor (H_2_O) and a 5th gas (α) used to model additives such as helium or other anaesthetic gases. During an iteration of the model, the flow (*f*) of air to or from an alveolar compartment *i* at time $t_{k}$is determined by the following equation:

$f_{i}(t_{k})=\frac{\left( p_{v}(t_{k})- p_{i}\left( t_{k} \right) \right)}{\left( \text{R}_{\text{u}}+\text{R}_{\text{A, }i} \right)}$ $\mathrm{for} i=1,\ldots,N_{A}$ (1)

where $p_{v}(t_{k})$ is the pressure at the mouth at $(t_{k})$, $p_{i}\left( t_{k} \right)$ is the pressure in the alveolar compartment $i$ at $(t_{k})$, R_u_ is the constant upper airway resistance and R_A,_ *_i_* is the bronchial inlet resistances of the alveolar compartment $i$. $N_{A}$is the total number of alveolar compartments (for the results in this paper, $N_{A}$ = 100). The total flow of air entering the SD at time $t_{k}$ is calculated by

$f_{SD}(t_{k}) = \sum_{i=1}^{N_{A}} f_{i}(t_{k})$ (2)

During the inhaling phase,$f_{SD}\geq0$, while in the exhaling phase$f_{SD}<0$. During gas movement in the SD, the fractions of gases in the layer $l$ of the SD,$F_{l,} (l =1,\ldots, N_{SD}$) is updated based on the composition of the total flow,$f_{SD}$, and the current composition of$F_{l,}$. If $f_{SD}$ ≥ 0, then air starts filling from the top layer ($l$ = 1) to the bottom layer ($l= N_{SD}$); and vice versa for $f_{SD}$< 0.

The volume of gas $\text{x}$, in the $i^{th}$ alveolar compartment ($\text{v}_{i\text{, x}}$), is given by:

$v_{i, x}(t_{k}) = \left\{ \begin{aligned} v_{i, x}\left( t_{k-1} \right)-f_{i}\left( t_{k} \right)\cdot\frac{v_{i, x}\left( t_{k-1} \right)}{v_{i}{(t}_{k})} Exhaling \\ v_{i, x}(t_{k-1}) +f_{i}(t_{k}) \cdot F_{N_{SD}}(t_{k}) Inhaling \end{aligned} \right.$ $\mathrm{for} i=1,\ldots,N_{A}$ (3)

In (3), x is any of the five gases (O_2_, N_2_, CO_2_, H_2_O or α). The total volume of the $i^{th}$ alveolar compartment, $v_{i}$ is the sum of the volume of the five gases in the compartment.

$v_{i}(t_{k})= v_{i,O2}(t_{k})+ v_{i,N2}(t_{k})+v_{i,CO2}(t_{k})+v_{i,H2O}(t_{k})+v_{i,\alpha}(t_{k})$ (4)

For the alveolar compartments, the tension at the centre of the alveolus and at the alveolar capillary border is assumed to be equal. The respiratory system has an intrinsic response to low oxygen levels in blood which is to restrict the blood flow in the pulmonary blood vessels, known as hypoxic pulmonary vasoconstriction (HPV). This is modelled as a simple function, resembling the stimulus response curve suggested by Marshall (9), and is incorporated into the simulator to gradually constrict the blood vessels as a response to low alveolar oxygen tension. The atmospheric pressure is fixed at 101.3 kPa and the body temperature is fixed at 37.2°C.

At each $t_{k}$, equilibration between the alveolar compartment and the corresponding capillary compartment is achieved iteratively by moving small volumes of each gas between the compartments until the partial pressures of these gases differ by <1% across the alveolar-capillary boundary. The process includes the nonlinear movement of O_2_ and CO_2_ across the alveolar capillary membrane during equilibration.

In blood, the total O_2_ content (C_O2_) is carried in two forms, as a solution and as oxyhaemoglobin (saturated haemoglobin):

$\text{C}_{\text{O2}}(t_{k})=\text{S}_{\text{O2}}(t_{k-1})\cdot Huf\cdot\text{Hb + }\text{P}_{\text{O2}}(t_{k-1})\cdot O_{2sol}$ (5)

In this equation, S_O2_ is the haemoglobin saturation, *Huf* is the Hufner constant, $\text{Hb}$ is the haemoglobin content and $O_{2sol}$ is the O_2_ solubility constant. The following pressure-saturation relation, as suggested by (10) to describe the O_2_ dissociation curve, is used in this model:

$\text{S}_{\text{O2}}(t_{k}) =\left( \left( \left( \text{P}_{\text{O2}}^{3}(t_{k-1})+150\cdot\text{P}_{\text{O2}}(t_{k-1}) \right)^{-1}\times23400 \right)+1 \right)^{-1}$ (6)

$\text{S}_{\text{O2}}$ is the saturation of the haemoglobin in blood and $\text{P}_{\text{O2}}$is the partial pressure of oxygen in the blood. As suggested by (11), $\text{P}_{\text{O2}}$has been determined with appropriate correction factors in base excess BE, temperature T and pH (7.5005168 = pressure conversion factor from kPa to mmHg):

$\text{P}_{\text{O2}}(t_{k})=7.5006\text{168 ∙ }\text{P}_{\text{O2}}(t_{k-1}) \cdot{10}^{\left[ 0.48\left( \text{pH}(t_{k-1})\text{-7.4} \right)-0.024\left( \text{T-37} \right)-0.0013\cdot\text{BE} \right]}$ (7)

The CO_2_ content of the blood (C_CO2_) is deduced from the plasma CO_2_ content (C_CO2plasma_) (12) by the following equation:

$\text{C}_{\text{CO2}}(t_{k})= \text{C}_{\text{CO2plasma}}(t_{k-1}) \cdot\left[ 1-\frac{0.0289\cdot\text{Hb}}{\left( 3.352-0.456 . \text{S}_{\text{O2}}(t_{k}) \right)\cdot\left( 8.142-\text{pH}(t_{k-1}) \right)} \right]$ (8)

where $\text{S}_{\text{O2}}$ is the O_2_ saturation, $\text{Hb}$ is the haemoglobin concentration and pH is the blood pH level. The coefficients were determined as a standardized solution to the McHardy version of Visser’s equation (13), by iteratively finding the best fit values to a given set of clinical data. The value of $\text{C}_{\text{CO2plasma}}$ is deduced using the Henderson-Hasselbach logarithmic equation for plasma C_CO2_ (14)_:_

$\text{C}_{\text{CO2plasma}}{(t}_{k})=2.226\cdot s_{CO2} \cdot\text{P}_{\text{CO2}}(t_{k-1}) \left( 1+ {10}^{\left( \text{pH}(t_{k-1}) \text{ – pK'} \right)} \right)$ (9)

where $s_{CO2}$ is the plasma CO_2_ solubility coefficient and $\text{pK'}$ is the apparent pK (acid dissociation constant of the CO_2_ bicarbonate relationship). $\text{P}_{\text{CO2}}$ is the partial pressure of CO_2_ in plasma and ‘2.226’ refers to the conversion factor from millimoles per liter to ml/100ml. (14) gives the equations for $s_{CO2}$ and $\text{pK'}$ as:

$s_{CO2}\text{= 0.0307 + 0.0057 ∙ }\left( 37-\text{T} \right)\text{ + 0.00002}{\cdot\left( 37-\text{T} \right)}^{2}$ (10)

$\text{pK' = 6.086 +0.042 ∙ (7.4 - pH}(t_{k-1}) \text{) + }\left( 38-\text{T} \right) \cdot\left( 0.00472+\left( 0.00139- \left( 7.4-\text{pH}(t_{k-1}) \right) \right) \right)$ (11)

$\text{P}_{\text{CO2}}\left( t_{k} \right)$is determined by incorporating the standard Henry’s law and the $s_{CO2}$(the CO_2_ solubility coefficient above). For pH calculation, the Henderson Hasselbach and the Van Slyke equation (15) are combined. Below is the derivation of the relevant equation. The Henderson-Hasselbach equation (governed by the mass action equation (acid dissociation)) states that:

$\text{pH = pK + log }\left( \frac{bicarbonateconcentration}{carbonicacidconcentration} \right)$ (12)

Substituting pK=6.1 (under normal conditions) and the denominator $(0.225 \cdot\text{P}_{\text{CO2}})$ (acid concentration being a function of CO_2_ solubility constant 0.225 and P_CO2_ (in kPa)) gives:

$\text{pH}{(t}_{k})\text{ = 6.1 + }\log\left( \frac{\text{HCO}_{\text{3}}(t_{k-1})}{0.225 \cdot\text{P}_{\text{CO2}(t_{k})}} \right)$ (13)

For a given pH, base excess (BE), and haemoglobin content (Hb), HCO_3_ is calculated using the Van-Slyke equation, as given by(15):

$\text{HCO}_{\text{3}}{(t}_{k})= \left( \left( 2.3 \times\text{Hb}+7.7 \right)\times\left( \text{pH}(t_{k})-7.4 \right) \right)+ \frac{\text{BE}}{\left( 1-0.023 \times\text{Hb} \right)}+ 24.4$ (14)

The capillary blood is mixed with arterial blood using the equation below which considers the anatomical shunt ($Sh)$ with the venous blood content of gas x ($\text{C}_{\text{v, x}})$, the non-shunted blood content from the pulmonary capillaries ($\text{C}_{\text{cap, x}}$), arterial blood content $\text{(}\text{C}_{\text{a, x}})$, the arterial volume $\text{(v}_{a})$ and the cardiac output (CO).

$\text{C}_{\text{a, x}}(t_{k})=\frac{\text{CO}(t_{k})\text{ ∙ }\left( Sh \cdot\text{C}_{\text{v, x}}(t_{k}) + \left( 1-Sh \right) \cdot\text{C}_{\text{cap, x}}(t_{k}) \right)+ \text{C}_{\text{a, x}}(t_{k})\cdot\left( \text{v}_{a}(t_{k})- \text{CO}(t_{k}) \right)}{\text{v}_{a}(t_{k})}$ (15)

The peripheral tissue model consists of a single tissue compartment, acting between the peripheral capillary and the active tissue (undergoing respiration to produce energy). The consumed O_2_ (V_O2_) is removed and the produced CO_2_ (V_CO2_) is added to this tissue compartment. As per the alveolar equilibration, peripheral capillary gas partial pressures reach equilibrium with the tissue compartment partial pressures, with respect to the nonlinear movement of O_2_ and CO_2_. Metabolic production of acids, other than carbonic acid via CO_2_ production, is not modelled. After peripheral tissue equilibration of gases, the venous calculations of partial pressures, concentrations and pH calculations are done using comparable equations as above.

A simple equation of renal compensation for acid base disturbance is incorporated. The base excess (BE) of blood under normal conditions is zero. BE increases by 0.1 per time slice if pH falls below 7.36 (to compensate for acidosis) and decreases by 0.1 per time slice if pH rises above 7.4 (alkalosis).

Each alveolar compartment has a unique and configurable alveolar compliance, alveolar inlet resistance, vascular resistance, extrinsic (interstitial) pressure and threshold opening pressure. For the $i$^th^ compartment of *N* alveolar compartments, the pressure $p_{i}$ is determined by:

$p_{i}{(t}_{k})=$ $S_{i}\left( v_{i}{(t}_{k})-V_{c} \right)^{2}+P_{ext,i}v_{i}{(t}_{k})+ P_{INSP}\mathrm{for}i=1,\ldots,N_{A}$ (16)

where

$S_{i}=k_{i}{N_{A}}^{2}/200000$ and $V_{c}=0.2V_{FRC}/N_{A}$

Equation (16) determines the alveolar pressure $p_{i}$ (as the pressure above atmospheric in cmH_2_O) for the $i$^th^ compartment of *N_A_* number of alveolar compartments for the given volume of alveolar compartment, $v_{i}(t)$ in millilitres. The alveolar compartments are arranged in parallel and interact with the series dead space with respect to the movement of gases. The use of the square of the difference between $v_{i}$ and *Vc* causes alveolar pressure to increase at volumes below *Vc*, leading to exhalation and a tendency to “snap shut” (mathematical note: the pressure with respect to volume is thus a U-shaped curve) (16).

$P_{ext}$ (per alveolar unit, in cmH_2_O) represents the *effective net pressure* generated by the sum of the effects of factors outside each alveolus that act to distend that alveolus; positive components include the outward pull of the chest wall, and negative effects include the compressive effect of interstitial fluid in the alveolar wall. Incorporating $P_{ext}$ in the model allows us to replicate the situation of alveolar units that have less structural support or that have interstitial oedema, and thus have a greater tendency to collapse. A negative value of $P_{ext}$ indicates a scenario where there is compression from outside the alveolus causing collapse. The parameter $S_{i}$ is a scalar that determines the intra-alveolar pressure for a given volume (with respect to a constant collapsing volume$V_{c}$) and is dependent on the parameter$k$. The units of $S_{i}$ are cmH_2_O ml^-2^. $P_{INSP}$, which represents the pressure generated by the respiratory muscles acting on the lung, is described in the next section. Finally, $V_{c}$ is defined as a “constant collapsing volume” at which the alveolus tends to empty (through Laplace effects) and represents a fundamental mechanical property of tissue and surfactant (16). $V_{FRC}$ is the end expiratory volume of the lungs.

The effect of the three parameters on the volume–pressure relationship of the alveolar compartments can be observed in Figure S1.2.


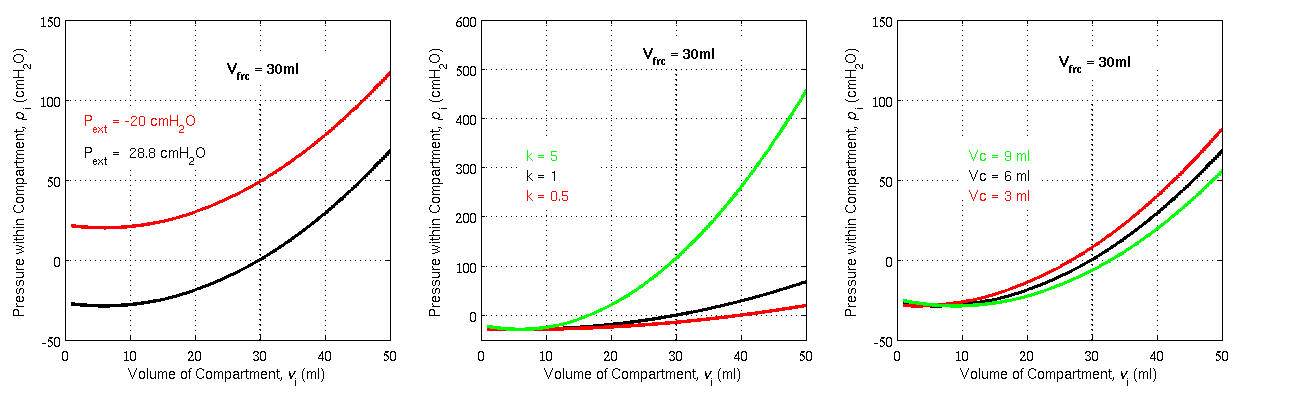


Figure S1.2 The effect of varying the parameters of Equation (18) on the pressure volume relationship of the model ($\boldsymbol{P}_{\boldsymbol{INSP}}$ = 0) and under mechanical ventilation

The nominal values for ($P_{ext,i}$ , $S_{i}$ ) have been determined such that at the end of expiration, the alveolar pressure within the compartment is also equal to zero, i.e. at an pre-set value of functional residual capacity.

We consider each of the parameters mentioned above ($P_{ext,i}$,$S_{i}$) to be different yet essential components for representing a diseased lung that affect the volume pressure relationship of the alveolar compartments. For example, for a given volume$v_{i}$, increasing$S_{i}$ increases the corresponding alveolar pressure of the alveolar compartment. When compared to another compartment with a lower$S_{i}$, a larger pressure gradient would be needed to drive air into the compartment; thus effectively the compartment will be behaving as a stiffer lung unit.

Decreasing $P_{ext,i}$ increases the alveolar pressure such that the pressure gradient (especially during exhaling) forces the air out of the alveolar compartment until the volume of the compartment collapses ($v_{i}$ = 0 ml). Note that, in effect, the parameters are influencing the resting volume of the compartments (when the alveolar pressure,$p_{i}$, is equal to zero).

In the model, the airway resistance $R_{aw}$is determined by the following equation for N parallel compartments:

| $\frac{1}{R_{aw}}=\frac{1}{R_{U}+R_{A,1}}+\frac{1}{R_{U}+R_{A,2}}+\cdots+\frac{1}{R_{U}+R_{A,N_{A}}}, \text{for}\text{ }i=1, \ldots,N_{A}$ | (17) |
| --- | --- |

where $R_{A,i}$is the bronchial inlet resistance of the $i^{th}$ compartment, which is defined by:

$R_{A,i}=m_{i}R_{A0}$

where $R_{A0}$corresponds to the default bronchial inlet resistance of an alveolar compartment. $R_{A0}$ is set to ${10}^{-7}\times N_{A}$ and $R_{U}$ is set to $2\times{10}^{-5}\times N_{A}$ (the inlet resistance is higher for a model with more compartments as the volume of each compartment decreases) for a healthy lung, giving a resistance of 0.00002 kPa.min.ml^-1^ for 100 compartments. $m_{i}$ is a coefficient of the airway resistance, representing a dynamic change in airway resistance and is determined by the equation:

$m_{i}=\left\{ \begin{aligned} 1, &t_{o,i}\leq0 \\ {10}^{10}, t_{o,i}>0 \end{aligned} \right.\text{for}i=1, \ldots,N_{A}$ (18)

where,

$t_{o, i}=\left\{ \begin{aligned} t_{o,i}-t , p_{trachea}\geq\text{TOP}_{\text{i}} \\ \tau_{c,i}, p_{trachea}<\text{TOP}_{\text{i}} \end{aligned}\text{ for}i=1, \ldots,N_{A} \right.$ (19)

$p_{trachea}$ is the pressure in the trachea and $TOP_{i}$ is a value between 5 and 50 cmH_2_O for the $i^{th}$ alveolar compartment. Additionally, a threshold opening pressure (TOP) at low lung volumes needs to be attained for a collapsed alveolar unit to open. Recruitment is a time dependent process, with different airways recruiting at different times, once the threshold pressure has been achieved (17, 18). The equations within the model are solved iteratively as a discretised system. Each iteration represents a physiological time slice of *t* (10 ms). The time-dependent recruitment phenomenon is achieved in the model by the introduction of a parameter$t_{o}$. For collapsed compartments, $t_{o}$ is set to $\tau_{c}$ which represents the time it could take for collapsed alveoli to open after a threshold pressure is reached. Once $p_{trachea}\geq\text{TOP}_{\text{i }}$is satisfied, the counter $t_{o}$decrements during every iteration, and triggers the opening of the airway ($m_{i}$= 1) as $t_{o}\leq0$. Otherwise $m_{i}$ is set to a high value (10^10^) to represent a collapsed airway.

$N_{A}$ (the number of alveolar compartments) is fixed and set by the user (i.e. they do not change during a simulation). Therefore, during a simulation, $m_{i}$, chiefly represents the relatively small changes in inlet resistance during tidal ventilation. Furthermore, $R_{B0}$are also preset and fixed, and do not change during the simulation. The only change in airway resistance which is dynamic is $m_{i}$ which is dependent on the volume$v_{i}$at time${(t}_{k})$.

Finally, the pulmonary vascular resistance PVR is determined by

| $\frac{1}{PVR}=\frac{1}{R_{V,1}}+\frac{1}{R_{V,2}}+\cdots+\frac{1}{R_{V,N_{A}}}, \text{for}\text{ }i=1, \ldots,N_{A}$ | (20) |
| --- | --- |

where the resistance for each compartment $R_{V,i}$is defined as

| $R_{V,i}=\delta_{Vi}R_{V0}$ | (21) |
| --- | --- |

$R_{V0}$ is the default vascular resistance for the compartment with a value of $160\cdot N_{A}$ dynes s cm^-5^ min^-1^, and $\delta_{Vi}$ is the vascular resistance coefficient, used to implement the effect of hypoxic pulmonary vasoconstriction.

The net effect of these components of the simulation is that the defining, clinical features of ARDS may be observed in the model: alveolar gas-trapping (with intrinsic PEEP), collapse-reopening of alveoli (with gradual reabsorption of trapped gas if re-opening does not occur), limitation of expiratory flow etc.

| **Parameter** | **Size** | **Ranges/Values** | |
| --- | --- | --- | --- |
|  |  | Open alveolar units | Collapsed alveolar units |
| P_ext,_*_i_* (cmH_2_O) | 100 | [-4.8,-6.1] | [2,10] |
| K*_i_* | 100 | [0.4,0.5] | [0.4,0.5] |
| TOP*_i_* (cmH_2_O) | 100 | [3.5,6.5] | [12,45] |
| Height | 1 | 174 | |
| Weight | 1 | 50+(0.91x(Height–152.4)) ^[22]^ | |
| RQ | 1 | 0.85 | |
| I:E | 1 | 1:2 | |
| VO2 (mL.min^-1^) | 1 | 4.2 x Weight ^[23]^ | |
| Hb (g.L^-1^) | 1 | 120 ^[24]^ | |
| Sh (%) | 1 | 2 ^[25]^ | |
| VD (mL) | 1 | 1.75 x Weight ^[26]^ | |

**Table S1 - List of the parameters required to calibrate the model, with their dimensions and allowable range of variation. P_ext,_*_i_* is the extrinsic pressure acting on compartments; K*_i_* is the stiffness of the compartments; TOP*_i_* is threshold opening pressure of the compartments; RQ is respiratory quotient; VO2 is total oxygen consumption; Hb is haemoglobin; VD is volume of anatomical dead space; Sh is anatomical shunt. Values of P_ext,_*_I_*, K*_i_*, and TOP*_i_* for each compartment are generated by randomly sampling from the specified range with a normal distribution, and are shown in Table S2.**

| **Pneumonitis Compartments** | | | | | **Thrombotic Compartments** | | | | | **Normal Compartments** | | | | | **Collapsed Compartments** | | | | |
| --- | --- | --- | --- | --- | --- | --- | --- | --- | --- | --- | --- | --- | --- | --- | --- | --- | --- | --- | --- |
| **#** | **TOP** | **P_ext_** | **K** | **R_V_ (**$\boldsymbol{\times1}\boldsymbol{0}^{\boldsymbol{3}}$**)** | **#** | **TOP** | **P_ext_** | **K** | **R_V_ (**$\boldsymbol{\times1}\boldsymbol{0}^{\boldsymbol{3}}$**)** | **#** | **TOP** | **P_ext_** | **K** | **R_V_ (**$\boldsymbol{\times1}\boldsymbol{0}^{\boldsymbol{3}}$**)** | **#** | **TOP** | **P_ext_** | **K** | **R_V_ (**$\boldsymbol{\times1}\boldsymbol{0}^{\boldsymbol{3}}$**)** |
| **1** | 5.92 | -5.62 | 0.47 | 16 | **21** | 6.48 | -6.01 | 0.46 | 80 | **31** | 6.15 | -5.65 | 0.44 | 16 | **93** | 39.26 | 2.38 | 0.43 | 3.2 |
| **2** | 5.35 | -5.76 | 0.46 | 16 | **22** | 6.13 | -5.26 | 0.45 | 80 | **32** | 5.48 | -6.04 | 0.46 | 16 | **94** | 28.58 | 4.79 | 0.45 | 3.2 |
| **3** | 4.72 | -5.24 | 0.44 | 16 | **23** | 4.12 | -5.36 | 0.46 | 80 | **33** | 3.58 | -5.61 | 0.44 | 16 | **95** | 12.51 | 3.73 | 0.46 | 3.2 |
| **4** | 4.91 | -5.50 | 0.46 | 16 | **24** | 4.13 | -5.86 | 0.45 | 80 | **34** | 6.37 | -5.77 | 0.45 | 16 | **96** | 30.76 | 5.44 | 0.44 | 3.2 |
| **5** | 3.52 | -5.76 | 0.45 | 16 | **25** | 6.33 | -5.25 | 0.44 | 80 | **35** | 5.44 | -5.15 | 0.46 | 16 | **97** | 33.16 | 7.20 | 0.46 | 3.2 |
| **6** | 4.81 | -5.02 | 0.44 | 16 | **26** | 5.50 | -5.63 | 0.45 | 80 | **36** | 3.77 | -5.08 | 0.47 | 16 | **98** | 19.96 | 3.87 | 0.47 | 3.2 |
| **7** | 3.98 | -5.95 | 0.45 | 16 | **27** | 3.77 | -5.48 | 0.48 | 80 | **37** | 4.35 | -5.13 | 0.44 | 16 | **99** | 22.68 | 4.25 | 0.46 | 3.2 |
| **8** | 4.91 | -5.73 | 0.47 | 16 | **28** | 4.97 | -4.92 | 0.45 | 80 | **38** | 5.80 | -5.47 | 0.46 | 16 | **100** | 43.96 | 8.45 | 0.45 | 3.2 |
| **9** | 5.95 | -5.94 | 0.44 | 16 | **29** | 3.96 | -5.20 | 0.47 | 80 | **39** | 4.09 | -5.59 | 0.46 | 16 |  |  |  |  |  |
| **10** | 5.14 | -5.57 | 0.46 | 16 | **30** | 6.13 | -5.44 | 0.43 | 80 | **40** | 3.57 | -4.96 | 0.44 | 16 |  |  |  |  |  |
| **11** | 3.62 | -5.60 | 0.44 | 16 |  |  |  |  |  | **41** | 3.90 | -5.33 | 0.45 | 16 |  |  |  |  |  |
| **12** | 4.34 | -5.27 | 0.45 | 16 |  |  |  |  |  | **42** | 4.55 | -6.01 | 0.47 | 16 |  |  |  |  |  |
| **13** | 3.66 | -5.91 | 0.43 | 16 |  |  |  |  |  | **43** | 5.57 | -5.63 | 0.48 | 16 |  |  |  |  |  |
| **14** | 4.55 | -5.16 | 0.44 | 16 |  |  |  |  |  | **44** | 3.79 | -5.79 | 0.47 | 16 |  |  |  |  |  |
| **15** | 3.92 | -4.96 | 0.46 | 16 |  |  |  |  |  | **45** | 5.00 | -4.94 | 0.44 | 16 |  |  |  |  |  |
| **16** | 4.58 | -5.23 | 0.45 | 16 |  |  |  |  |  | **46** | 3.90 | -5.42 | 0.46 | 16 |  |  |  |  |  |
| **17** | 4.11 | -5.47 | 0.45 | 16 |  |  |  |  |  | **47** | 5.38 | -4.92 | 0.44 | 16 |  |  |  |  |  |
| **18** | 4.37 | -5.06 | 0.43 | 16 |  |  |  |  |  | **48** | 5.13 | -5.36 | 0.46 | 16 |  |  |  |  |  |
| **19** | 5.75 | -5.49 | 0.44 | 16 |  |  |  |  |  | **49** | 5.31 | -5.50 | 0.47 | 16 |  |  |  |  |  |
| **20** | 5.87 | -5.19 | 0.43 | 16 |  |  |  |  |  | **50** | 5.91 | -5.88 | 0.47 | 16 |  |  |  |  |  |
|  |  |  |  |  |  |  |  |  |  | **51** | 5.07 | -5.31 | 0.43 | 16 |  |  |  |  |  |
|  |  |  |  |  |  |  |  |  |  | **52** | 3.80 | -5.84 | 0.46 | 16 |  |  |  |  |  |
|  |  |  |  |  |  |  |  |  |  | **53** | 5.54 | -5.15 | 0.45 | 16 |  |  |  |  |  |
|  |  |  |  |  |  |  |  |  |  | **54** | 5.96 | -4.90 | 0.45 | 16 |  |  |  |  |  |
|  |  |  |  |  |  |  |  |  |  | **55** | 4.63 | -5.94 | 0.45 | 16 |  |  |  |  |  |
|  |  |  |  |  |  |  |  |  |  | **56** | 3.50 | -5.70 | 0.44 | 16 |  |  |  |  |  |
|  |  |  |  |  |  |  |  |  |  | **57** | 3.55 | -5.67 | 0.45 | 16 |  |  |  |  |  |
|  |  |  |  |  |  |  |  |  |  | **58** | 4.28 | -5.76 | 0.48 | 16 |  |  |  |  |  |
|  |  |  |  |  |  |  |  |  |  | **59** | 6.33 | -4.88 | 0.45 | 16 |  |  |  |  |  |
|  |  |  |  |  |  |  |  |  |  | **60** | 4.70 | -5.59 | 0.48 | 16 |  |  |  |  |  |
|  |  |  |  |  |  |  |  |  |  | **61** | 3.95 | -5.46 | 0.43 | 16 |  |  |  |  |  |
|  |  |  |  |  |  |  |  |  |  | **62** | 4.41 | -5.53 | 0.46 | 16 |  |  |  |  |  |
|  |  |  |  |  |  |  |  |  |  | **63** | 4.00 | -5.54 | 0.47 | 16 |  |  |  |  |  |
|  |  |  |  |  |  |  |  |  |  | **64** | 4.62 | -5.09 | 0.44 | 16 |  |  |  |  |  |
|  |  |  |  |  |  |  |  |  |  | **65** | 5.49 | -5.84 | 0.43 | 16 |  |  |  |  |  |
|  |  |  |  |  |  |  |  |  |  | **66** | 5.70 | -5.22 | 0.46 | 16 |  |  |  |  |  |
|  |  |  |  |  |  |  |  |  |  | **67** | 3.90 | -5.37 | 0.44 | 16 |  |  |  |  |  |
|  |  |  |  |  |  |  |  |  |  | **68** | 4.25 | -5.06 | 0.44 | 16 |  |  |  |  |  |
|  |  |  |  |  |  |  |  |  |  | **69** | 6.43 | -5.16 | 0.44 | 16 |  |  |  |  |  |
|  |  |  |  |  |  |  |  |  |  | **70** | 5.61 | -4.93 | 0.46 | 16 |  |  |  |  |  |
|  |  |  |  |  |  |  |  |  |  | **71** | 4.78 | -5.75 | 0.43 | 16 |  |  |  |  |  |
|  |  |  |  |  |  |  |  |  |  | **72** | 3.93 | -5.31 | 0.45 | 16 |  |  |  |  |  |
|  |  |  |  |  |  |  |  |  |  | **73** | 4.88 | -5.59 | 0.44 | 16 |  |  |  |  |  |
|  |  |  |  |  |  |  |  |  |  | **74** | 5.30 | -5.02 | 0.45 | 16 |  |  |  |  |  |
|  |  |  |  |  |  |  |  |  |  | **75** | 5.76 | -5.29 | 0.43 | 16 |  |  |  |  |  |
|  |  |  |  |  |  |  |  |  |  | **76** | 4.06 | -5.02 | 0.48 | 16 |  |  |  |  |  |
|  |  |  |  |  |  |  |  |  |  | **77** | 5.97 | -5.27 | 0.45 | 16 |  |  |  |  |  |
|  |  |  |  |  |  |  |  |  |  | **78** | 4.19 | -5.42 | 0.47 | 16 |  |  |  |  |  |
|  |  |  |  |  |  |  |  |  |  | **79** | 6.22 | -5.52 | 0.43 | 16 |  |  |  |  |  |
|  |  |  |  |  |  |  |  |  |  | **80** | 6.36 | -5.53 | 0.47 | 16 |  |  |  |  |  |
|  |  |  |  |  |  |  |  |  |  | **81** | 5.00 | -5.81 | 0.48 | 16 |  |  |  |  |  |
|  |  |  |  |  |  |  |  |  |  | **82** | 4.50 | -5.83 | 0.43 | 16 |  |  |  |  |  |
|  |  |  |  |  |  |  |  |  |  | **83** | 4.08 | -5.45 | 0.45 | 16 |  |  |  |  |  |
|  |  |  |  |  |  |  |  |  |  | **84** | 3.74 | -5.45 | 0.45 | 16 |  |  |  |  |  |
|  |  |  |  |  |  |  |  |  |  | **85** | 3.99 | -5.69 | 0.43 | 16 |  |  |  |  |  |
|  |  |  |  |  |  |  |  |  |  | **86** | 5.40 | -5.49 | 0.48 | 16 |  |  |  |  |  |
|  |  |  |  |  |  |  |  |  |  | **87** | 5.78 | -5.94 | 0.45 | 16 |  |  |  |  |  |
|  |  |  |  |  |  |  |  |  |  | **88** | 4.96 | -5.99 | 0.48 | 16 |  |  |  |  |  |
|  |  |  |  |  |  |  |  |  |  | **89** | 4.30 | -5.56 | 0.44 | 16 |  |  |  |  |  |
|  |  |  |  |  |  |  |  |  |  | **90** | 5.36 | -5.72 | 0.45 | 16 |  |  |  |  |  |
|  |  |  |  |  |  |  |  |  |  | **91** | 6.02 | -5.36 | 0.44 | 16 |  |  |  |  |  |
|  |  |  |  |  |  |  |  |  |  | **92** | 4.09 | -4.85 | 0.43 | 16 |  |  |  |  |  |

**Table S2 - List of each single parameter value required to calibrate the model. P_ext_ is the extrinsic pressure acting on compartments; K is the stiffness of the compartments; TOP is threshold opening pressure of the compartments and R_V_ is the vascular resistance of the compartments.**

# Section 2: Data for the population of 10 patients at baseline

| **Patient** | **Collapsed (%)** | **Microthrombosis (%)** | **Disrupted (%)** | **PaO2 (mmHg)** | **PaCO2 (mmHg)** | **SaO2 (%)** | **VT (mL)** | **EELV (mL)** | **DP (cmH2O)** | **Power (J/min)** | **P_MIN_ (cmH2O)** | **Phys Shunt (%)** | **Phys VD (mL)** | **VD/VT** | **C_rs (mL/cmH2O)** | **C_L (mL/cmH2O)** | **Ppl_swing (cmH2O)** | **Ptp_swing (cmH2O)** | **Total Strain** |
| --- | --- | --- | --- | --- | --- | --- | --- | --- | --- | --- | --- | --- | --- | --- | --- | --- | --- | --- | --- |
| **1** | 8 | 10 | 20 | 51.2 | 52.2 | 84 | 487.6 | 1678.4 | 7.744 | 1.57 | -12.4 | 49.5 | 188.5 | 0.387 | 63 | 111.9 | 12 | 4.7 | 0.291 |
| **2** | 9 | 10 | 20 | 48 | 52.5 | 81 | 489.9 | 1663.2 | 7.855 | 1.62 | -12.6 | 51.9 | 192.5 | 0.393 | 62.4 | 110 | 12.2 | 4.8 | 0.295 |
| **3** | 9 | 8 | 18 | 50.8 | 50.7 | 83 | 489.9 | 1663.2 | 7.856 | 1.62 | -12.6 | 49.7 | 184.7 | 0.377 | 62.4 | 110 | 12.2 | 4.8 | 0.295 |
| **4** | 10 | 10 | 20 | 45.3 | 53.9 | 78 | 484.5 | 1645.9 | 7.855 | 1.61 | -12.6 | 54.1 | 193.4 | 0.399 | 61.7 | 107.9 | 12.2 | 4.8 | 0.294 |
| **5** | 10 | 8 | 17 | 48.8 | 51.4 | 82 | 484.5 | 1645.9 | 7.854 | 1.61 | -12.6 | 51.2 | 183.4 | 0.379 | 61.7 | 107.9 | 12.2 | 4.8 | 0.294 |
| **6** | 11 | 10 | 20 | 42.9 | 54.6 | 75 | 486.6 | 1629.9 | 7.969 | 1.65 | -12.8 | 56.1 | 197.7 | 0.406 | 61.1 | 106 | 12.4 | 4.9 | 0.299 |
| **7** | 7 | 10 | 20 | 54.8 | 51.3 | 86 | 489 | 1695.4 | 7.688 | 1.55 | -12.3 | 47 | 185.8 | 0.38 | 63.6 | 113.9 | 11.9 | 4.6 | 0.288 |
| **8** | 6 | 10 | 20 | 59.2 | 50.3 | 89 | 490.3 | 1712 | 7.631 | 1.54 | -12.2 | 44.4 | 183 | 0.373 | 64.3 | 116 | 11.8 | 4.6 | 0.286 |
| **9** | 5 | 10 | 20 | 64.8 | 50 | 91 | 487.5 | 1727.6 | 7.515 | 1.49 | -12 | 41.5 | 179.4 | 0.368 | 64.9 | 118 | 11.6 | 4.5 | 0.282 |
| **10** | 5 | 11 | 22 | 60.5 | 51.2 | 89 | 487.5 | 1727.6 | 7.515 | 1.49 | -12 | 43.6 | 185.1 | 0.38 | 64.9 | 118 | 11.6 | 4.5 | 0.282 |

**Table S3: Data for the population of 10 patients at baseline**

# Section 3: Modelling spontaneous ventilation

The pressure at the airway opening (e.g. mouth) is relatively constant at atmospheric pressure, while the alveolar pressure value varies during inspiration and expiration. In a healthy individual, the active contraction of the respiratory muscles during inspiration generates a negative alveolar pressure relative to the atmospheric pressure. The variable $P_{INSP}$, which represents the pressure generated by the respiratory muscles acting on the lung, is modelled as a piecewise function as described in (20) and adapted from (21). The function consists of a parabolic profile during the inspiration phase of the respiratory cycle, representing the progressive increase in pressure exerted by the respiratory muscles, followed by an exponential profile during the expiration phase of the respiratory cycle, characterizing the passive relaxation of the muscles. During a single respiratory cycle, $P_{INSP}$ at time $t_{k}$, is calculated as

$$P_{INSP}\left( t_{k} \right)= \left\{ \begin{aligned} \frac{-P_{MIN}}{T_{I}.T_{E}}.{t_{k}}^{2}+\frac{P_{MIN}.T}{T_{I}.T_{E}}.t_{k} t_{k} \in\left\lfloor0,T_{I} \right\rfloor\\ \frac{P_{MIN}}{1-e^{-\frac{T_{E}}{\tau}}}.\left( {e^{-\frac{\left( {{(t}_{k}-T}_{I} \right)}{\tau}}-e}^{- \frac{T_{E}}{\tau}} \right) t_{k} \in\left\lfloor T_{I}, T \right\rfloor\end{aligned} \right.$$

$P_{INSP}$ decreases from zero to its minimum end-inspiratory value ($P_{MIN}$) (i.e., maximum effort) during inspiration and returns to zero at end of expiration. $T$ is calculated from the set respiratory rate, RR, ($T$ = 60/RR). $T_{I}$ and $T_{E}$ are the duration of inspiration and expiration, such that ($T$ = $T_{I}+ T_{E}$). $T_{I}$ is calculated from ($T_{I}=T*DC)$ where $DC$ is the duty cycle,set to 0.33. $\tau$ is the time constant of the expiratory profile and is set to Te/RR.

The resultant alveolar pressure $p_{i}$ depends on $P_{INSP}$, as well as on the pressures of the gases within the compartment, the stiffness of the compartment ($S_{i}$), and parameter $P_{ext}$ which represents extrinsic pressures acting on the compartment.

# Section 4: Modelling Covid-19 Acute Respiratory Failure

The computational simulator has been configured to represent a number of pathophysiological mechanisms proposed to underlie COVID-19 AHRF. The resulting model replicates the available clinical data (severe hypoxemia combined with preserved pulmonary compliance and gas volumes in ‘Type L’ ARDS patients). Baseline settings represent a typical 70 kg patient with body mass index of 24 kg/m^2^.

To model the well-preserved lung compliance seen in type L COVID-19 patients, 8% of the alveolar compartments are modelled as collapsed by increasing the corresponding threshold opening pressures (TOP) and the extrinsic pressures ($P_{ext}$). HPV is normally incorporated in the simulator via a mathematical function, based on the stimulus response curve suggested in (9); to simulate the hypothesised disruption of HPV in CARDS this function has been disabled. Hyper-perfusion was modelled by decreasing the vascular resistance to the collapsed compartments. Pneumonitis is represented by disrupting the gas exchange in 20% of the alveolar compartments, by removing those compartments from the “for-loop” that calculates gas-exchange in the relevant model function. The effects of microthrombi are implemented by increasing the vascular resistance to perfusion in 10% of the compartments.

# Section 5: Effect of Respiratory effort on compartmental volumes

Figs S5.1 and S5.2 show the effect of increased respiratory effort on the distribution of maximum compartmental volumes. The model consists of 100 independently configured gas-exchanging compartments. Compartments 93 to 100 are collapsed at baseline, at higher respiratory effort some of these compartments are partially recruited. As shown, at the higher values of tidal volume and respiratory rate, the proportion of compartments experiencing higher maximum volumes is significantly increased.

**Figure S5.1 Distribution of maximum compartmental volumes in the model. The model consists of 100 independently configured gas-exchanging compartments. Compartments 93 to 100 are collapsed at baseline, at higher VT some of these compartments are partially recruited.**

**Figure S5.2: Distribution of maximum compartmental volumes in the model. The model consists of 100 independently configured gas-exchanging compartments. (a) VT = 7 ml/kg, RR = 14 b/min, (b) VT = 10 ml/kg, RR = 14 b/min, (c) VT = 7 ml/kg, RR = 20 b/min, (d) VT = 10 ml/kg, RR = 20 b/min, (e) VT = 7 ml/kg, RR = 30 b/min, (f) VT = 10 ml/kg, RR = 30 b/min.**

# References

1. Hardman J, Aitkenhead A. Estimation of alveolar deadspace fraction using arterial and end-tidal CO2: a factor analysis using a physiological simulation. Anaesthesia and intensive care. 1999;27(5):452.

2. Hardman J, Bedforth N. Estimating venous admixture using a physiological simulator. British journal of anaesthesia. 1999;82(3):346-9.

3. Hardman J, Bedforth N, Ahmed A, Mahajan R, Aitkenhead A. A physiology simulator: validation of its respiratory components and its ability to predict the patient's response to changes in mechanical ventilation. British journal of anaesthesia. 1998;81(3):327-32.

4. Hardman JG, Aitkenhead AR. Validation of an original mathematical model of CO2 elimination and dead space ventilation. Anesthesia & Analgesia. 2003;97(6):1840-5.

5. Hardman J, Wills J. The development of hypoxaemia during apnoea in children: a computational modelling investigation. British journal of anaesthesia. 2006;97(4):564-70.

6. Das A, Gao Z, Menon P, Hardman J, Bates D. A systems engineering approach to validation of a pulmonary physiology simulator for clinical applications. Journal of The Royal Society Interface. 2011;8(54):44-55.

7. McCahon R, Columb M, Mahajan R, Hardman J. Validation and application of a high-fidelity, computational model of acute respiratory distress syndrome to the examination of the indices of oxygenation at constant lung-state. British journal of anaesthesia. 2008;101(3):358-65.

8. Hardman JG, Al-Otaibi HM. Prediction of arterial oxygen tension: validation of a novel formula. American journal of respiratory and critical care medicine. 2010;182(3):435-6.

9. Marshall BE, Clarke WR, Costarino AT, Chen L, Miller F, Marshall C. The dose-response relationship for hypoxic pulmonary vasoconstriction. Respir Physiol. 1994;96(2-3):231-47.

10. Severinghaus JW. Simple, accurate equations for human blood O2 dissociation computations. J Appl Physiol Respir Environ Exerc Physiol. 1979;46(3):599-602.

11. Severinghaus JW. Blood gas calculator. J Appl Physiol. 1966;21(3):1108-16.

12. Douglas AR, Jones NL, Reed JW. Calculation of whole blood CO2 content. J Appl Physiol (1985). 1988;65(1):473-7.

13. McHardy GJ. The relationship between the differences in pressure and content of carbon dioxide in arterial and venous blood. Clin Sci. 1967;32(2):299-309.

14. Kelman GR, Nunn JF. Nomograms for correction of blood Po2, Pco2, pH, and base excess for time and temperature. J Appl Physiol. 1966;21(5):1484-90.

15. Siggaard-Andersen O. The van Slyke equation. Scand J Clin Lab Invest Suppl. 1977;146:15-20.

16. Lachmann B. Open up the lung and keep the lung open. Intensive Care Med. 1992;18(6):319-21.

17. Hickling KG. The pressure-volume curve is greatly modified by recruitment. A mathematical model of ARDS lungs. Am J Respir Crit Care Med. 1998;158(1):194-202.

18. Bates JH, Irvin CG. Time dependence of recruitment and derecruitment in the lung: a theoretical model. J Appl Physiol (1985). 2002;93(2):705-13.

19. Crotti S, Mascheroni D, Caironi P, Pelosi P, Ronzoni G, Mondino M, et al. Recruitment and derecruitment during acute respiratory failure: a clinical study. Am J Respir Crit Care Med. 2001;164(1):131-40.

20. Mecklenburgh JS, Mapleson WW. Ventilatory assistance and respiratory muscle activity. 2: Simulation with an adaptive active ("aa" or "a-squared") model lung. Br J Anaesth. 1998;80(4):434-9.

21. Albanese A, Cheng L, Ursino M, Chbat NW. An integrated mathematical model of the human cardiopulmonary system: model development. Am J Physiol Heart Circ Physiol. 2016;310(7):H899-921. 22. MacDonald JJ, Moore J, Davey V, Pickering S, Dunne T. The weight debate. J Intensive Care Soc. 2015;16(3):234-238. doi:10.1177/1751143714565059 23. S. A. McLellan, MB ChB BSc MRCP FRCA, T. S. Walsh, MB ChB BSc MRCP FRCA MD, Oxygen delivery and haemoglobin, Continuing Education in Anaesthesia Critical Care & Pain, Volume 4, Issue 4, August 2004, Pages 123–126, https://doi.org/10.1093/bjaceaccp/mkh033 24. Dean L. Blood Groups and Red Cell Antigens [Internet]. Bethesda (MD): National Center for Biotechnology Information (US); 2005. Table 1, Complete blood count. Available from: https://www.ncbi.nlm.nih.gov/books/NBK2263/table/ch1.T1/ 25. SAID SI, BANERJEE CM. Venous admixture to the pulmonary circulation in human subjects breathing 100 per cent oxygen. J Clin Invest. 1963 Apr;42(4):507-15. doi: 10.1172/JCI104739. PMID: 13976084; PMCID: PMC289310. 26. Numa, Andrew & Newth, Christopher. (1996). Anatomic dead space in infants and children. Journal of applied physiology (Bethesda, Md. : 1985). 80. 1485-9. 10.1152/jappl.1996.80.5.1485.
